# The association of rod curvature with postoperative outcomes in patients undergoing posterior lumbar interbody fusion for spinal stenosis: a retrospective case–control study

**DOI:** 10.1186/s12891-023-06404-y

**Published:** 2023-04-18

**Authors:** Lin Han, Hongdao Ma, Qisheng Li, Jincan Yuan, Haisong Yang, Yuchen Qin, Xuhua Lu

**Affiliations:** 1 Department of Orthopaedics, Shanghai Changzheng Hospital, Second Military Medical University, Shanghai, 200003 China; 2 Department of Orthopaedics, Third Affiliated Hospital of Naval Medical University, Shanghai, 200433 China; 3 Department of Health Statistics, Second Military Medical University, Shanghai, 200003 China

**Keywords:** Lumbar surgery, Posterior lumbar interbody fusion, Rod bending, Rod contouring, Rod curvature

## Abstract

**Background:**

Restoration of sagittal balance is a crucial consideration in posterior lumbar interbody fusion (PLIF) surgery and adverse postoperative outcomes are associated with inadequate restoration of sagittal alignment. However, there remains a shortage of substantial evidence regarding the effect of rod curvature on both sagittal spinopelvic radiographic parameters and clinical outcomes.

**Method:**

A retrospective case–control study was conducted in this study. Patient demographics (age, gender, height, weight and BMI), surgical characteristics (number of fused levels, surgical time, blood loss and hospital stay) and radiographic parameters (lumbar lordosis [LL], sacral slope [SS], pelvic incidence [PI], pelvic tilt [PT], PI-LL, Cobb angle of fused segments [Cobb], rod curvature [RC], Posterior tangent angle of fused segments [PTA] and RC-PTA) were analyzed.

**Results:**

Patients in the abnormal group had older mean age and suffered more blood loss than those in the normal group. In addition, RC and RC-PTA were significantly lower in the abnormal group compared to the normal group. Multivariate regression analysis revealed that lower age (OR = 0.94; 95% CI: 0.89–0.99; *P* = 0.0187), lower PTA (OR = 0.91; 95% CI: 0.85–0.96; *P* = 0.0015) and higher RC (OR = 1.35; 95% CI: 1.20–1.51; *P* < 0.0001) were related to higher odds of better surgical outcomes. The receiver operating characteristic curve analysis showed that the ROC curve (AUC) for predicting outcomes of surgery by RC classifier was 0.851 (0.769–0.932).

**Conclusions:**

In patients who underwent PLIF surgery for lumbar spinal stenosis, those who had a satisfactory postoperative outcome tended to be younger, had lower blood loss, and higher values of RC and RC-PTA compared to those who had poor recovery and required revision surgery. Additionally, RC was found to be a reliable predictor of postoperative outcomes.

**Supplementary Information:**

The online version contains supplementary material available at 10.1186/s12891-023-06404-y.

## Introduction

Lumbar spinal stenosis affects approximately 103 million people worldwide [1]. Various treatment modalities include oral medications, physical therapy, injections, bracing, and surgical treatment [2]. Surgical treatment such as decompression alone, decompression with fusion, and percutaneous decompression is considered effective when treating severe spinal stenosis, and routine care after surgery also plays a role in improving prognosis [3].

Posterior lumbar interbody fusion (PLIF) was first reported to be successfully performed in 1940s and later promoted by the combination with pedicle screw techniques from 1980s [4]. PLIF relieves pressure on the spinal cord and nerve roots and provides support and stability through internal fixation instruments. Posterior screw-rod instrumentation is a well-established internal fixation system to provide sufficient support and restores sagittal balance during PLIF surgery. The rod curvature is suggested to be consistent with physiological LL to maintain spinal balance. Restoration of sagittal balance is a key issue in lumbar fusion surgery. Adverse postoperative outcomes are many times ascribed to inadequate restoration of sagittal alignment [5]. The SRS-Schwab Adult Spinal Deformity Classification reported that sagittal vertical axis (SVA), pelvic tilt (PT), and the difference between pelvic incidence (PI) and lumbar lordosis (LL) (i.e. PI-LL) were significantly associated with health-related quality of life (HRQOL) [6]. Yigor et al*.* presented Global Alignment and Proportion (GAP) score, including PI, sacral slope (SS), LL, Global Tilt and age factors, to predict prognosis in patients with spinal deformity [7].

Moufid et al*.* presented the mismatch analysis index (MAI) to describe the relationship between rod bending and post-operative LL in L3-L5 lumbar fusion [8]. Surgeons usually perform the rod bending procedure using a French bender according to their experience or preferences in clinal practice. Hence, the subjective behavior of rod bending brings about differences in rod curvature, which might have an impact on the clinical results. It was reported that rod flattening might lead to low back pain, flatback syndrome, acceleration of adjacent segment degeneration, and sagittal malalignment [9]. But there is still a lack of solid evidence on the correlation between rod curvature and clinical outcomes. Moreover, the appropriate degree of rod curvature is also needed to be further evaluated, although Shi et al*.* reported that the Cobb angle could be used as a reference for rod contouring for patients with thoracolumbar fractures [10].

Here, a retrospective clinical study was conducted to compare clinical and radiographic characteristics between patients who underwent posterior lumbar interbody fusion (PLIF) with satisfactory postoperative results and those who had poor prognoses resulted from adjacent segment degeneration and required revision surgery. We hypothesized that rod curvature was associated with adverse surgical results.

## Material and methods

### Subjects

Patients who underwent PLIF surgery with the diagnosis of lumbar spinal stenosis were collected from the medical record data of Shanghai Changzheng Hospital from January 2015 to June 2021 with institutional review board (IRB) approval and informed consent (Approval No.2014SL031). We identified 133 patients who underwent PLIF. Among these 133 patients, normal and abnormal groups were further extracted by their inclusion criteria respectively. The normal group (*n* = 53) included the ones with satisfactory postoperative recovery of function and no obvious symptoms for at least 3 years, and excluded the ones (*n* = 11) with satisfactory but less 3 years. The abnormal group (*n* = 44) consisted of those who had poor recovery from the previous PLIF within 3 years, and required revision surgery for the reason of adjacent segment degeneration. Cases (*n* = 25) requiring surgery for infection or other reasons were excluded. Exclusion criteria were as follows: (1) apparent spinal deformity such as kyphosis, lordosis, and scoliosis; (2) lumbar spondylolisthesis with unsatisfactory operative reduction; (3) thoracolumbar vertebral fracture; (4) other diseases that cause obvious spinal instability. (5) follow-up period < 3 years after PLIF surgery.

### Description of PLIF surgery

All the patients underwent PLIF with the same technique by the same surgical team. The surgical technique of PLIF was described as follows: the spine was approached through a longitudinal midline incision of the back and bilateral erector spinae are stripped off the lamina at the corresponding surgical site. After the insertion of pedicle screws, laminectomy was performed to allow the visualization of dural sac and nerve roots. The stenosis of spinal canal and nerve root canal was relieved and the pressured nerve was decompressed completely. Then the disc and adjacent endplates were removed and replaced with an interbody cage with bone graft.

### Patient demographic and surgical characteristics

**Fig. 1:**
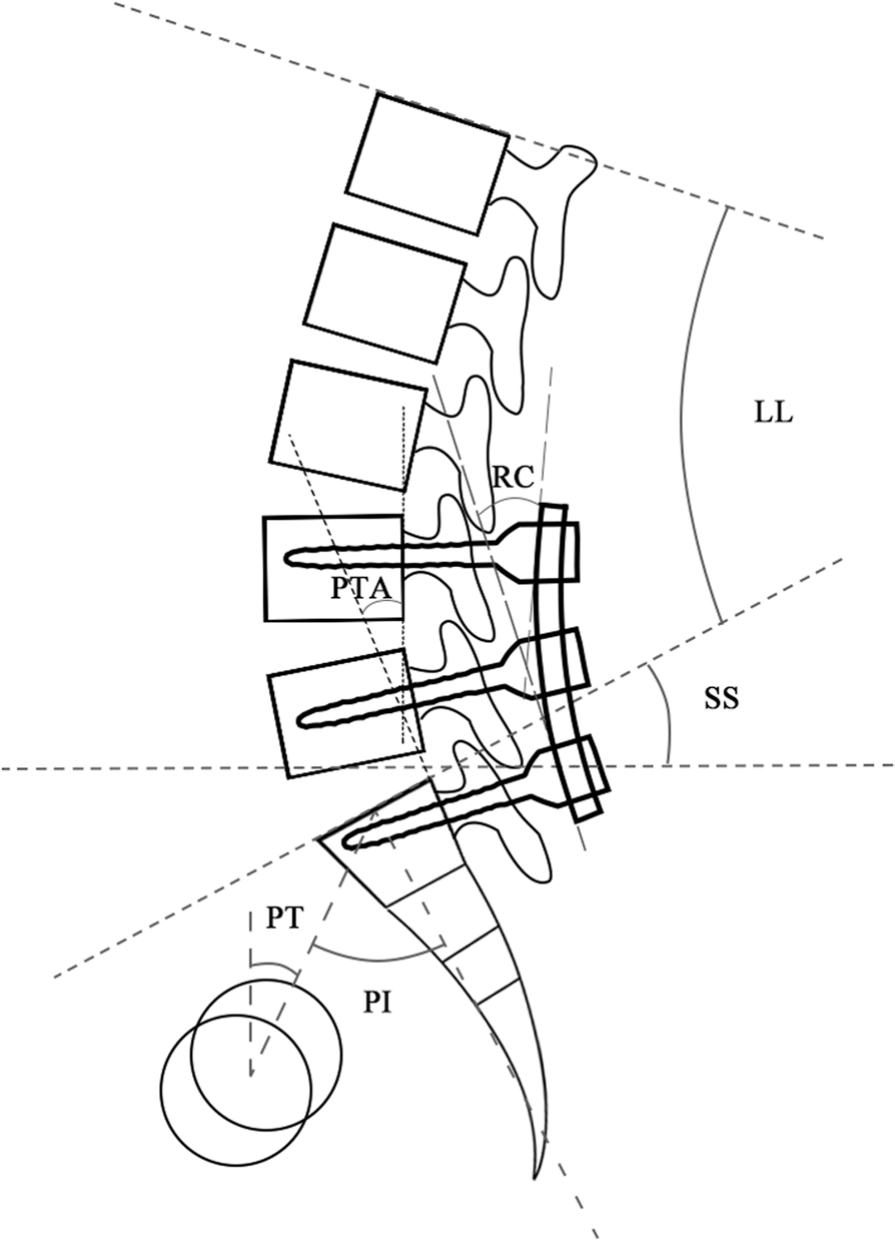
Description of radiographic parameters in the lateral radiograph. Abbreviation: *LL*, lumbar lordosis; *PT*, pelvic tilt; *PI*, pelvic incidence; *SS*, sacral slope; *RC*, rod curvature; *PTA*, posterior tangent angle of fused segments

Patient demographics (age, gender, height, weight and BMI) and surgical characteristics (number of fused levels, surgical time, blood loss and hospital stay) were collected from the initial inpatient medical records in both groups. Lateral views of the lumbar spine were collected from the last follow-up in the Normal group and the latest examination before revision surgery in the Abnormal group. The radiographic parameters (Fig. 1) were measured as follows: (1) LL: the angle between the superior endplates of L1 and S1. (2) SS: the angle between S1 superior plate and the horizontal line. (3) PI: the angle between the perpendicular line of S1 superior endplate and the line connecting the midpoint of S1 superior endplate to the midpoint of two femoral head centers. (4) PT: the angle between the plumb line and the line connecting the midpoint of the S1 superior endplate to the midpoint of two femoral head centers. (5) PI-LL: the difference between PI and LL. (6) Cobb angle of fused segments (Cobb): the angle between the superior and inferior endplates of the fused vertebral bodies. (7) rod curvature (RC): intersecting tangent angle at each end of the convex rod. (8) Posterior tangent angle of fused segments (PTA): intersecting tangent angle on the cranial and caudal segments of the fused vertebral bodies. (9) RC-PTA: RC minus PTA.

### Statistics analysis

Continuous variables were expressed as mean (SD) and compared using t-test or Kruskal–Wallis rank sum test conditional on the distribution of the variables. Categorical variables were compared using the χ2 test or Fisher exact test. Considering that PI-LL mismatch was a widely accepted risk factor for operative disability [11], patients were categorized into two types (absolute value of PI-LL > 10° and ≤ 10°) and analyzed to evaluate the effect of PI-LL mismatch on surgical outcomes. A multivariate stepwise logistic regression was used to determine independent predictors for surgical effect (abnormal/normal). Receiver operating characteristic curve (ROC) analysis was used to calculate the optimal cutoff value for RC classifier that was determined by maximizing the Youden index (ie, sensitivity + specificity—1). The performance of RC classifier for predicting surgery outcomes was assessed by the sensitivity, specificity, predictive values and likelihood ratio values. Analyses were performed by SAS 9.4 (SAS Institute, Cary, NC) and R 3.6.3 (R Development Core Team) software. *P* < 0.05 was defined as a significant difference.

## Results

**Fig. 2:**
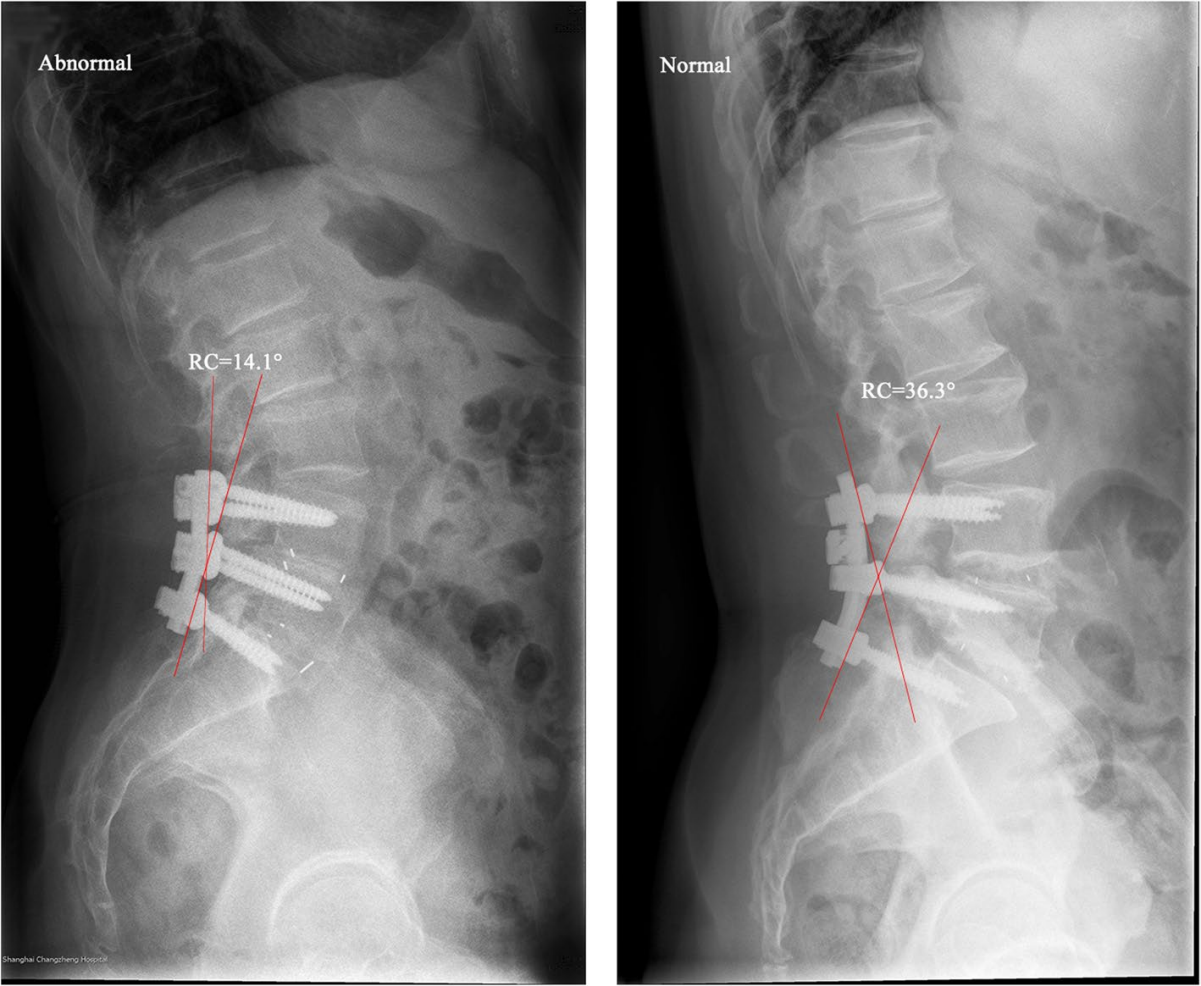
Typical cases show rod curvature differences between abnormal and normal groups

In this retrospective study, 44 and 53 patients were identified in abnormal and normal groups, respectively. A comparison of patient characteristics was shown in Table 1. Patients in the abnormal group had older mean age (59.91 ± 9.78 versus 52.72 ± 12.39, *P* = 0.002) and suffered more blood loss (419.32 ± 275.18 ml versus 343.40 ± 275.62 ml, *P* = 0.033) than patients in the normal group. No significant difference was observed between the two groups in aspects of gender, height, weight, BMI, surgical time and hospital stay. As for radiographic parameters, RC and RC-PTA were significantly lower in the abnormal group compared with the normal group (10.71° ± 7.98° versus 22.04° ± 7.9°, *P* < 0.001; -12.23° ± 11.22° versus 0.10° ± 10.21°, *P* < 0.001; (Fig. 2). But there was no difference in LL, PT, PI, SS, PI-LL, Cobb and PTA. In addition, PI-LL mismatch or not didn’t show a significant difference in abnormal and normal groups (*P* = 0.719).

**Table 1:** Comparison of patient characteristics in abnormal and normal groups

Variables	Total (*n* = 97)	Abnormal (*n* = 44)	Normal (*n* = 53)	P	Statistic
Age, Mean ± SD	55.98 ± 11.79	59.91 ± 9.78	52.72 ± 12.39	0.002	1586.5
Gender, n (%)				1	0
Male	51 (52.58)	23 (52.27)	28 (52.83)		
Female	46 (47.42)	21 (47.73)	25 (47.17)		
Height, Mean ± SD	1.66 ± 0.08	1.67 ± 0.07	1.66 ± 0.09	0.766	1207.5
Weight, Mean ± SD	68.41 ± 12.08	66.98 ± 10.06	69.59 ± 13.52	0.278	-1.092
BMI, Mean ± SD	24.58 ± 3.22	23.99 ± 2.77	25.07 ± 3.49	0.093	-1.697
Number of Fused Levels, n (%)				0.391	Fisher
1	54 (55.67)	21 (47.73)	33 (62.26)		
2	35 (36.08)	20 (45.45)	15 (28.3)		
3	5 (5.15)	2 (4.55)	3 (5.66)		
4	2 (2.06)	1 (2.27)	1 (1.89)		
5	1 (1.03)	0 (0.00)	1 (1.89)		
Surgical Time, Mean ± SD	2.73 ± 0.77	2.89 ± 0.87	2.59 ± 0.66	0.068	1412.5
Blood Loss, Mean ± SD	377.84 ± 276.60	419.32 ± 275.18	343.40 ± 275.62	0.033	1453
Hospital Stay, Mean ± SD	7.53 ± 2.85	7.52 ± 2.92	7.53 ± 2.83	0.994	1167.5
LL, Mean ± SD	49.01 ± 11.5	47.06 ± 12.77	50.63 ± 10.16	0.138	-1.498
PT, Mean ± SD	15.2 ± 7.4	15.02 ± 7	15.36 ± 7.78	0.82	-0.228
PI, Mean ± SD	50.23 ± 10.3	49.82 ± 10.28	50.58 ± 10.41	0.721	-0.358
SS, Mean ± SD	35.03 ± 8.15	34.80 ± 9.31	35.22 ± 7.13	0.81	-0.241
PI-LL, Mean ± SD	1.22 ± 10.21	2.76 ± 10.61	-0.05 ± 9.79	0.182	1.344
PI-LL, n (%)				0.719	0.129
> 10	28 (28.87)	14 (31.82)	14 (26.42)		
≤ 10	69 (71.13)	30 (68.18)	39 (73.58)		
Cobb, Mean ± SD	27.87 ± 17.32	29.51 ± 21.63	26.52 ± 12.76	0.791	1203
RC, Mean ± SD	16.90 ± 9.72	10.71 ± 7.98	22.04 ± 7.90	< 0.001	-6.991
PTA, Mean ± SD	22.39 ± 13.18	22.94 ± 14.14	21.94 ± 12.44	0.948	1175.5
RC-PTA, Mean ± SD	-5.49 ± 12.29	-12.23 ± 11.22	0.10 ± 10.21	< 0.001	465

^a^The difference between two groups were tested by Kruskal–Wallis rank sum test as the distribution of the variable didn’t fully conform to normal distribution

*Abbreviation*: *LL* Lumbar lordosis, *PT* Pelvic tilt, *PI* Pelvic incidence, *SS* Sacral slope, *PI-LL* The difference between PI and LL, *Cobb* Cobb angle of fused segments, *RC* Rod curvature, *PTA* Posterior tangent angle of fused segments, *RC-PTA* RC minus PTA

Multivariate regression analysis revealed that lower age (OR = 0.94; 95% CI: 0.89–0.99; *P* = 0.0187), higher RC (OR = 1.35; 95% CI: 1.20–1.51) and lower PTA (OR = 0.91; 95% CI: 0.85–0.96) were related to higher odds of better surgical outcome (Table 2). ROC curve analysis showed that RC classifier was a pretty good classifier for predicting postoperative outcomes. The area under the ROC curve (AUC) for predicting outcomes of surgery by RC classifier was 0.851 (0.769–0.932) and the optimal RC cut-off value was 13.70 (Supplementary Material 1).

**Table 2:** Multivariate stepwise logistic regression analysis of characteristics on surgical effects between abnormal and normal groups

	Estimate	Standard Error	Wald χ^2^	P	OR (95% CI)
Intercept	1.33	1.54	0.75	0.3863	
Age	-0.07	0.03	5.53	0.0187	0.94(0.89–0.99)
RC	0.30	0.06	25.92	< .0001	1.35(1.20–1.51)
PTA	-0.10	0.03	10.13	0.0015	0.91(0.85–0.96)

*Abbreviation*: *RC* Rod curvature, *PTA* Posterior tangent angle of fused segments

^a^Baseline patient characteristics, such as Age, Gender, Height, Weight, Number of Fused Levels, Surgical Time, Blood Loss, Hospital Stay, LL (lumbar Lordosis), PI-LL (the difference between PI and LL), Cobb (Cobb angle of fused segments), RC (rod curvature) and PTA (Posterior tangent angle of fused segments), were included in the initial logistic model in which both the significance level for entry and the significance level for staying were set to 0.1

Under this optimal cut-off value, the sensitivity was 0.89, specificity was 0.75, the positive predictive value was 0.81, negative predictive value was 0.85, positive likelihood ratio was 3.55 and negative likelihood ratio was 0.16 (Table 3). The AUC for predicting outcomes of surgery by RC-PTA classifier was 0.801 (0.713, 0.888) and the optimal RC cut-off value was -1.30 (Supplementary Material 2). Under this optimal cut-off value, the sensitivity was 0.60, specificity was 0.89, the positive predictive value was 0.86, negative predictive value was 0.65, positive likelihood ratio was 5.31 and negative likelihood ratio was 0.45.

**Table 3:** The performance of rod curvature (RC) for predicting postoperative outcomes under the optimal cutoff value

	Estimate
Optimal Cutoff	13.70
Sensitivity	0.89
Specificity	0.75
Positive predictive value	0.81
Negative predictive value	0.85
Positive likelihood ratio	3.55
Negative likelihood ratio	0.16

## Discussion

Current research demonstrated that insufficient RC and mismatch between RC and PTA (RC-PTA) were associated with adverse surgical outcomes when patients underwent PLIF surgery, even if the lumbar physiological parametersfollowed the recommendations from previous literature such as PI-LL < 11°and PT < 22° [6, 12, 13]. In this study, a new parameter called RC-PTA was introduced to assess the discrepancy between the contouring of rods and the curvature of the lumbar spine. The PTA method, which was proposed by Harrison et al*.* [14] and shown to be more suitable than the Cobb method for evaluating lumbar lordosis, was also used. Furthermore, this study revealed that RC was a reliable predictor of postoperative outcomes. From our findings, it also should be noted that the revision group had older age which can potentially lead to poorer outcomes, and slightly increased blood loss which might mean either a more complex fusion was performed, or patients had more bony or ligamental damage. Both variables could potentially confound the results.

To date, there are few studies about rod contouring in PLIF surgery. Shi et al*.* reported that 4° to 8° greater than the cobb angle of fused segments was suggested as the optimal reference angle for rod contouring in patients with thoracolumbar fractures [10]. Their study emphasized that the accuracy of rod bending angle was important for spinal sagittal balance and prevention of adjacent disc degeneration. Zhao et al*.* performed a retrospective analysis to evaluate the effect of contoured versus straight rods on the radiographic and clinical outcomes in patients undergoing the minimally invasive transforaminal lumbar interbody fusion (MIS-TLIF) at L4/5 level, and found contoured or straight rods had no statistical difference in global spinopelvic parameters and clinical outcomes in 5 years follow-up [15]. One possible explanation for this opposite finding relative to our results is that stripping off spinal muscles attached to the lamina in PLIF surgery could reduce the stability of posterior spine components and the muscular decollement might result in the risk of postoperative back pain. Besides, several works of literature focused on rod contouring in long segmental thoracolumbar deformity correction. Salmingo et al*.* demonstrated that preoperative implant rod curvature was relevant to postoperative rod deformation degree in adolescent idiopathic scoliosis (AIS) [16]. Yan et al*.* found that the mismatch between proximal rod contouring and proximal spinal curve might be a predisposing risk factor for postoperative proximal junctional kyphosis (PJK) in degenerative scoliosis [17]. Moreover, Wang et al*.* revealed that over 5° difference between proximal junctional angle and rod contouring angle (PJA-RCA) was a risk factor for PJK in Lenke I and II adolescent idiopathic scoliosis (AIS) patients [18]. Lafage et al*.* also indicated that proper rod contouring played a critical role in reducing the risk of PJK [19].

Polyaxial screws are user-friendly to rod installation during posterior spinal surgery, which allows mutual adaptation of screw heads and rods in certain deviations. In single-segment PLIF surgery with a small LL, some surgeons may prefer to use unbent rods, as noted in a previous study [15]. However, although polyaxial screws can partially compensate for LL and make the surgery more convenient, there may be a mismatch between the screw heads and the rod that could affect the amount of lordosis and clinical outcomes [9]. In certain cases, rod contouring alone may not be sufficient to achieve the desired LL [8]. A biomechanical study by Paik et al*.* reported that if a rod persuasion device was used to reduce the rod-pedicle screw mismatch, the peak pullout strength of the screw and failure energy significantly decreased, especially in the osteoporotic spine [20]. A finite element study also demonstrated that the correction of this mismatch could result in high forces at the screws and high stress in adjacent tissue and inferior spinal segments [21]. Combined with our results, it is suggested that surgeons should pay extra attention to rod contouring procedures to make the rods fit with corresponding spinal curvature.

As the realignment of sagittal balance was correlated with adequate rod curvature [22], reflecting the critical role of the intraoperative rod bending step, precise rod bending techniques are introduced to optimize this work. According to Wanivenhaus et al*.*'s study, the utilization of augmented reality technology in rod bending during surgery could lead to a reduction of 20% in surgery time and improvement in the accuracy of rod length [23]. In spine deformity surgery, patient-specific rods were also utilized to help achieve satisfactory sagittal alignment [9, 24]. Ohba et al*.* introduced a computer-assisted rod bending system to decrease the occurrence of screw pull-out and loosening after surgery [25]. Moreover, precise rod bending techniques could prevent repetitive bending operations that could increase the risk of metal fatigue [26].

This study confirmed that rod contouring procedure had an impact on clinical outcomes in patients with lumbar spinal stenosis undergoing PLIF surgery. Smaller RC and RC-PTA mismatches were found in patients who previously received PLIF and required lumbar revision surgery. Further, the RC was demonstrated to be a pretty good classifier for predicting postoperative outcomes. Our research revealed the importance of rod curvature relative to spinal alignment in lumbar pedicle screw-rod fixation surgery. Nevertheless, some limitations also should be acknowledged in our study. First, this study is a retrospective case–control study which is hard to avoid selection bias. Therefore, future studies with large sample size from multiple centers are needed to verify our results. Second, patient prognosis was influenced by various contributors. The duration and intensity of daily activities and the severity of lumbar degeneration varies among different patients, which were not taken into account in current research. Third, surgical-related factors such as the range and extent of surgical depression, sagittal spine re-alignment, the orientation of pedicle screws, might have an impact on patient outcomes. Further biomechanical experiments and randomized double-blind controlled clinical trials are recommended to validate the findings.

## Conclusions

The study revealed significant differences in age, RC and RC-PTA between lumbar spinal stenosis patients who underwent PLIF surgery with satisfactory outcome and those who had poor prognosis resulted from adjacent segment degeneration and required revision surgery. RC was demonstrated to be a pretty good indicator for predicting postoperative outcomes.

## Supplementary Information

**Additional file 1:** **Supplement Digital Content 1.** Summary receiver operating characteristic curvesfor RC for predicting postoperative outcomes. **Supplement Digital Content 2.** Summary receiver operatingcharacteristic curves for RC-PTA for predicting postoperative outcomes.

## Data Availability

The datasets generated and analyzed during the current study are not publicly available due to patient privacy concerns but are available from the corresponding author on reasonable request.
