# Correction: The association of rod curvature with postoperative outcomes in patients undergoing posterior lumbar interbody fusion for spinal stenosis: a retrospective case–control study

**DOI:** 10.1186/s12891-023-06532-5

**Published:** 2023-05-20

**Authors:** Lin Han, Hongdao Ma, Qisheng Li, Jincan Yuan, Haisong Yang, Yuchen Qin, Xuhua Lu

**Affiliations:** 1 Department of Orthopaedics, Shanghai Changzheng Hospital, Second Military Medical University, Shanghai, 200003 China; 2 Department of Orthopaedics, Third Affiliated Hospital of Naval Medical University, Shanghai, 200433 China; 3 Department of Health Statistics, Second Military Medical University, Shanghai, 200003 China

**Correction: BMC Musculoskelet Disord 24, 304 (2023)**

10.1186/s12891-023-06404-y

Following publication of the original article [1], the authors identified an error in the order of affiliations (Affiliation 1 and 2 were transposed).

The order of affiliations has been updated above and the original article [1] has been corrected.
