# Supplementary material for: The association of rod curvature with postoperative outcomes in patients undergoing posterior lumbar interbody fusion for spinal stenosis: a retrospective case–control study
Source: BMC Musculoskelet Disord. 2023 Apr 18;24:304. doi: 10.1186/s12891-023-06404-y (PMC10111816; doi:10.1186/s12891-023-06404-y)
Supplement: Supplementary file 1 — Additional file 1: Supplement Digital Content 1. Summary receiver operating characteristic curvesfor RC for predicting postoperative outcomes. Supplement Digital Content 2. Summary receiver operatingcharacteristic curves for RC-PTA for predicting postoperative outcomes. [file 12891_2023_6404_MOESM1_ESM.docx]

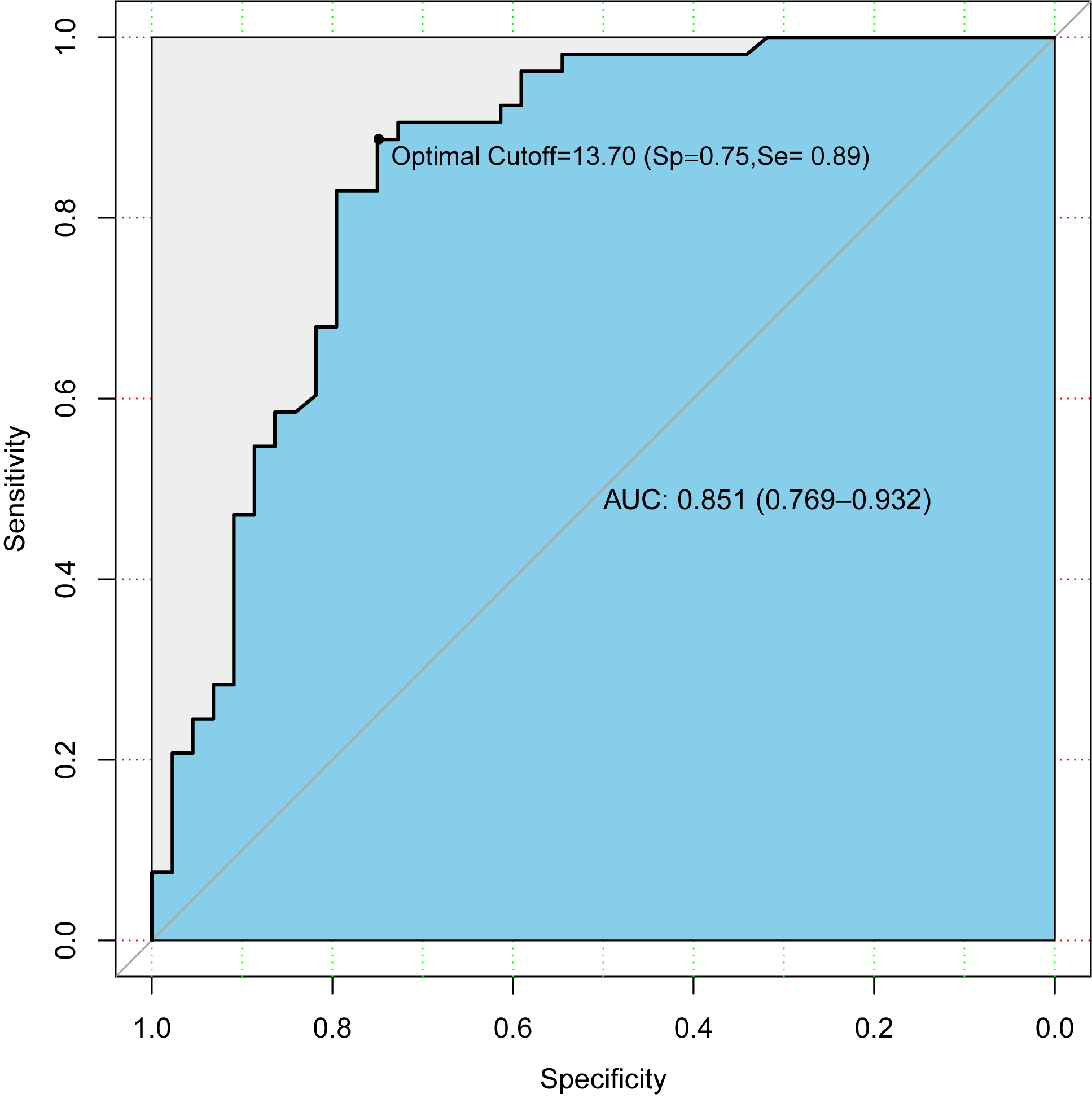


**Supplement Digital Content 1:** Summary receiver operating characteristic curves for RC for predicting postoperative outcomes.


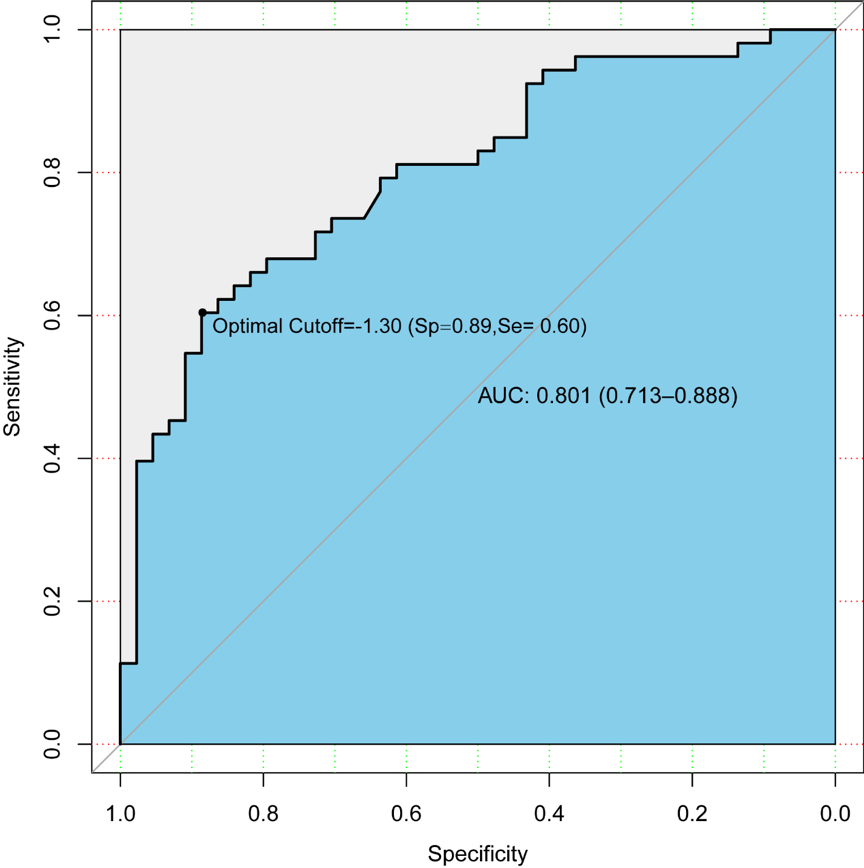


**Supplement Digital Content 2 :** Summary receiver operating characteristic curves for RC-PTA for predicting postoperative outcomes.
